# Driving factors of retention in care among HIV-positive MSM and transwomen in Indonesia: A cross-sectional study

**DOI:** 10.1371/journal.pone.0191255

**Published:** 2018-01-17

**Authors:** Adi Nugroho, Vicki Erasmus, Robert W. S. Coulter, Sushil Koirala, Oranuch Nampaisan, Wirastra Pamungkas, Jan Hendrik Richardus

**Affiliations:** 1 Department of Public Health, Erasmus MC, University Medical Center Rotterdam, Rotterdam, The Netherlands; 2 School of Public Health, Medical Faculty, Lambung Mangkurat University, Banjarbaru, Indonesia; 3 GWL-INA Network, Jakarta, Indonesia; 4 Department of Behavioral and Community Health Sciences, Graduate School of Public Health, University of Pittsburgh, Pittsburgh, Pennsylvania, United States of America; 5 Center for LGBT Health Research, Graduate School of Public Health, University of Pittsburgh, Pittsburgh, Pennsylvania, United States of America; 6 Asia Pacific Network of People Living with HIV/AIDS, Bangkok, Thailand; Katholieke Universiteit Leuven Rega Institute for Medical Research, BELGIUM

## Abstract

Little is known about the prevalence of and factors that influence retention in HIV-related care among Indonesian men who have sex with men (MSM) and transgender women (transwomen, or waria in Indonesian term). Therefore, we explored the driving factors of retention in care among HIV-positive MSM and waria in Indonesia. This cross-sectional study involved 298 self-reported HIV-positive MSM (n = 165) and waria (n = 133). Participants were recruited using targeted sampling and interviewed using a structured questionnaire. We applied a four-step model building process using multivariable logistic regression to examine how sociodemographic, predisposing, enabling, and reinforcing factors were associated with retention in care. Overall, 78.5% of participants were linked to HIV care within 3 months after diagnosis or earlier, and 66.4% were adequately retained in care (at least one health care visit every three months once a person is diagnosed with HIV). Being on antiretroviral therapy (adjusted odds ratio [AOR] = 6.00; 95% confidence interval [CI]: 2.93–12.3), using the Internet to find HIV-related information (AOR = 2.15; 95% CI: 1.00–4.59), and having medical insurance (AOR = 2.84; 95% CI: 1.27–6.34) were associated with adequate retention in care. Involvement with an HIV-related organization was associated negatively with retention in care (AOR = 0.47; 95% CI: 0.24–0.95). Future interventions should increase health insurance coverage and utilize the Internet to help MSM and waria to remain in HIV-related care, thereby assisting them in achieving viral suppression.

## Introduction

Men who have sex with men (MSM) and transgender women (transwomen) are disproportionately burdened by HIV infection worldwide. Compared to the general adult population, MSM and transgender people have 19- and 34-fold greater odds, respectively, of getting infected with HIV [1]. In Indonesia, HIV prevalence among MSM and “*waria*” (an Indonesian term for transwomen) is substantial: HIV prevalence rose from 5% in 2007 to 12% in 2011 among MSM, and was even higher among waria at 24% in 2007 and 23% in 2011 [2].

Given the high prevalence of HIV among MSM and waria, the Indonesia government currently supports a “Test and Treat” strategy. Such a strategy aims to initiate antiretroviral therapy (ART) for HIV-positive individuals who have a high chance of transmitting the virus to their sex partners (due to their high-risk behavior) regardless of their CD4 count [3], including MSM and waria. The success of this strategy is evaluated through the HIV treatment cascade, a system that monitors the number of individuals living with HIV who are linked to care, receive medical care, remain engaged in care; and are able to suppress the virus through treatment [4–6]. Immediate linkage to care after an HIV-positive diagnosis allows HIV-patients to receive appropriate counseling and treatment, which can prevent further HIV sequelae and transmission [7]. Both patients who are on antiretroviral therapy (ART) and those who are not should remain retained in care [8]. For those who are on ART, retention in care is necessary to ensure adherence, manage toxicities, and address treatment failure [8, 9]. For those not on ART, regular engagement in care allows doctors to monitor patients and provide prophylactic medication and ART initiation once indicated [10, 11]. These common clinical practices help patients attain and maintain viral suppression, which is the ultimate indicator for successful management of HIV infection [12] and can prevent sexual transmission of HIV infection [13–15].

Among HIV-positive Indonesians, generally, the proportion of HIV-positive individuals alive and receiving ART after one year of initiating treatment was less than 70% in 2011, with loss to follow-up from treatment at 19% [16]. In 2013, HIV treatment retention for 12, 24, and 60 months was 66%, 62%, and 44%, respectively [17]. These drop-out levels are comparable to the treatment retention of HIV-positive MSM and transwomen populations in other countries [18, 19]. However, specific data about how HIV-positive MSM and waria in Indonesia are linked to and retained in care is scarce. Because anti-gay and anti-trans stigma in Indonesia are high [20], linking and retaining these stigmatized groups to care is likely worse than that of HIV-positive individuals in general. Understanding the prevalence of retention in care among MSM and waria is therefore warranted.

Furthermore, studies examining factors that facilitate and create barriers to proper retention in HIV care for Indonesian MSM and waria can help inform future interventions that aim to increase retention in care. Prior studies in other countries indicate the following common barriers to engagement in HIV care among MSM: psychological burden of committing to HIV care; accessibility of HIV treatment; privacy and treatment concerns; perceived discrimination from healthcare workers; and lack of guidance and follow-up [21–24]. Barriers to ART access among transgender women primarily consist of fear of their HIV-positive status being revealed to others; HIV stigma within transgender communities themselves; rejection by family; social isolation, loss of subsistence income; and maltreatment within the healthcare system [25–27]. Having culturally appropriate and transgender-sensitive healthcare was a strong facilitator to engagement and retention in care [28].

Understanding how vulnerable populations are linked to and retained in HIV care is critical to achieve optimal clinical outcomes. However, factors that enable or inhibit retention in care among Indonesian MSM and waria have yet to be examined. To address these gaps in knowledge, this study aimed to describe retention in HIV care among HIV-positive MSM and waria in Indonesia. This study also investigated the driving factors of retention in care among the populations. We examined predisposing, enabling, and reinforcing factors, which are based on the PRECEDE-PROCEED Model [29], a theoretical model for evaluating psycho-sociological health behavior.

## Methods

### Study design and population

We analyzed cross-sectional baseline data from a study conducted by the Asia Pacific AIDS Positive Network (APN+), a regional network of people living with HIV from 11 countries in Asia and the Pacific. Data were collected by trained interviewers from 1,655 Indonesian people living with HIV from December 2012 to February 2013. In this paper we focus on the MSM and waria subsample (n = 298; 165 MSM and 133 waria). Participants were administered a validated questionnaire developed by the APN+. The original questionnaire was in English, and translated to Bahasa Indonesia. The questionnaire was then back-translated into English to ensure consistency and quality.

Participants were recruited using a targeted sampling method [30], a technique that has been widely used for sampling hard-to-reach groups. First, at least one person from each high-risk group with HIV (including MSM and waria) was selected to be a seed at each study site. These people were identified either through the contact of a local support group for people living with HIV or through a local community-based organization. Each seed was surveyed and then asked to recruit another participant to the study. Then this participant was surveyed and asked to recruit another participant to the study. This process was repeated until the desired sample size at each study site was reached.

Inclusion of the participants was based on the following criteria: 1) an HIV-positive individual residing in the study sites; 2) aged between 18–50 years; and 3) self-reported diagnosed with HIV infection at least three months prior to the date of interview. Participants were informed about the study objectives and the procedures prior to being surveyed. Those who voluntarily signed the informed consent form were then given the interviewer-administered survey. Study procedures were approved by the Research Ethics Board of Atma Jaya University Indonesia, Jakarta.

### Measures

The primary outcome measure in our analysis was retention in HIV care, which was measured using the following question: “After being HIV-positive, how frequently did you visit your doctor/nurse/health worker?” Response options included: once a week; once a month; once every 2–3 months; once every 4–6 months; only every 7–12 months; or only when I am sick). We dichotomized participants into having adequate and inadequate retention in care. We defined adequate retention as at least one health care visit every three months once a person is diagnosed with HIV. This definition is in accordance with guidelines and prior studies [8, 10, 31]. As additional information, we also measured linkage to care using the following question: “After diagnosis, how long did it take you to meet the doctor/nurse/health worker?” Response options consisted of: right after diagnosis, same day; number of years/months/days after diagnosis; or not visited yet.

To explore the determinants of retention in HIV care, we analyzed the impacts of three groups of influencing factors: predisposing, enabling, and reinforcing factors based on the PRECEDE-PROCEED model [29]. *Predisposing factors* included HIV treatment literacy, which was defined as the level of understanding on all aspects of ART, including types of ART drugs, ART side effects, treatment adherence, HIV drug resistance and other related topics. To measure this, an overall literacy score was applied using 25 “True” or “False” questions (Cronbach’s alpha = 0.90; higher scores indicate greater knowledge). The remaining predisposing factors were: disease history (e.g., in the past 6 months, did you suffer from any disease/health problem? yes versus no); ever diagnosed with TB (have you ever been diagnosed with TB after you were HIV+? yes versus no); alcohol drinking (do you currently drink alcohol? yes versus no); illicit drug use (have you ever used any illicit drugs? Yes versus no); smoking (do you smoke? yes versus no); and unsafe sex in the past 6 months (in the past 6 months, have you had sex with your spouse/someone other than your spouse? yes versus no); how frequently did you use a condom when you had sex with him/her?).

*Enabling factors* included having medical insurance (are you enrolled in any kind of health insurance program? yes versus no); and being a member of/affiliated to an HIV-related organization (are you a member of/affiliated to any HIV-related organization? yes versus no); internet use for HIV information (have you ever used internet to find HIV-related information? yes versus no), and amount of internet use (in the past 7 days, how many hours did you spend on the internet?).

*Reinforcing factors* included ART status (are you taking ART/HIV medicines now? yes versus no); disclosure to steady partner (have you ever disclosed your HIV+ status to your spouse? yes versus no); disclosure to individuals beyond family & steady partner (have you ever disclosed your HIV+ status to anyone except your spouse, a close family member, and your doctor? yes versus no); stigma & discrimination experience (in the last 12 months, how often have you been excluded from social events or activities; or been verbally insulted, harassed and/or threatened; or been physically assaulted; or been denied health services because of your HIV status?); and social support, which was measured using a validated 12 items scale [32]. These items addressed instrumental and emotional social support from family, friends, and significant other (e.g. “my family really tries to help me”; “I can count on my friends when things go wrong”; and “there is a special person with whom I can share my joys and sorrows”). Responses ranged from “strongly disagree” to “strongly agree” on a five-point scale (Cronbach’s alpha = 0.90; higher scores indicate greater social support). Sociodemographic characteristics were also measured. This included risk group (MSM or waria), age, education, income, and place of residence.

### Analyses

We analyzed data in Stata version 14.0 (StataCorp, College Station, Texas). We first examined differences between MSM and waria in sociodemographic characteristics, linkage to care, and retention in care, as well as predisposing, enabling, reinforcing factors using chi-square tests for categorical variables and independent t-test or Mann-Whitney U tests (when data were not normally distributed) for continuous variables. We then examined the associations of sociodemographic characteristics as well as predisposing, enabling, reinforcing factors with our primary outcome, retention in HIV care using univariate logistic regression models. When univariate p-values were less than 0.20, we included these factors in multivariable models. We fit multivariable logistic regression models to identify factors associated with retention in care. We used a four-step model building process, based on the PRECEDE-PROCEED Model [29]. We added factors cumulatively: first, we added sociodemographic characteristics (Model 1), followed by predisposing factors (Model 2), enabling factors (Model 3), and reinforcing factors (Model 4). Assumptions for logistic regression were met (i.e., non-collinearity, linearity in the logit, no outliers, independence of observations).

## Results

### Sociodemographic characteristics

Table 1 describes the sociodemographic characteristics of the sample. Compared to waria participants, MSM participants were significantly younger, were better educated, had a higher monthly income, and were less likely to have ever engaged in sex work.

**Table 1:** Sociodemographic characteristics of total sample, and by risk group.

			Risk group				
	Total		MSM		Waria^a^		
Characteristic	*N*	*%*	*n*	*%*	*n*	*%*	*p-value*
**Total**	298	100	165	55.4	133	44.6	
**Age, mean (SD)**	32.1	(7.4)	29.9	(6.2)	34.9	(7.8)	<0.001
**Highest education level**							
Primary school or lower	14	4.7	2	1.2	12	9.0	<0.001
Secondary school	63	21.4	3	1.8	60	45.1	
Higher secondary school	173	58.1	115	69.7	58	43.6	
College degree or above	48	16.1	45	27.3	3	2.3	
**Monthly income (in US dollars), median**	109.1	-	136.4	-	90.9	-	<0.000
**Relationship status**							
Single	231	77.5	129	78.2	102	76.7	0.171
Has HIV-negative partner	28	9.4	15	9.1	13	9.8	
Has HIV-positive partner	16	5.4	12	7.3	4	3.0	
Has partner with unknown HIV status	23	7.7	9	5.5	14	10.5	
**Area of residence**							
Rural area /small town	61	20.5	25	15.2	36	27.1	0.011
Large town/city	237	79.5	140	84.8	97	72.9	
**Recruitment site**							
Jakarta and surroundings	76	25.5	44	26.7	32	24.1	0.029
Bandung and surroundings	148	49.7	91	55.2	57	42.9	
Yogyakarta & Central Java	46	15.4	19	11.5	27	20.3	
City outside of Java Island	28	9.4	11	6.7	17	12.8	
**Sex work in lifetime**	94	31.5	10	6.1	84	63.2	<0.001
**Injection drug use in lifetime**	4	1.3	3	1.8	1	0.8	0.427

*Note*: P-values were derived from Chi-Square test for categorical variables, and independent t-tests or Mann-Whitney U test for continuous variables; SD = standard deviation;

^a^Waria = Indonesian term for transwomen.

### Linkage to and retention in HIV care

Table 2 indicates that the majority of respondents (78.5%) were linked to HIV care within 3 months after receiving their HIV diagnosis, and this did not differ between MSM and waria. Almost two-thirds (66.4%) of respondents had adequate retention in health care (i.e., they visited a healthcare facility at least once every three months since being diagnosed with HIV). Retention in HIV care was higher among MSM than waria participants (72.7% vs 58.7%, respectively, p < 0.05).

**Table 2:** Linkage to and retention in HIV care, and determinant factors on health care uptake among respondents, and by risk group.

			Risk group				
	Total		MSM		Waria^a^		
Variable	*N*	*%*	*n*	*%*	*n*	*%*	*p-value*
**Total**	298	100	165	55.4	133	44.6	
**Linkage to HIV care**							
≤ 3 months after diagnosis	234	78.5	132	80.0	102	76.7	0.152
4–6 months after diagnosis	7	2.3	1	0.6	6	4.5	
> 6 months after diagnosis	30	10.1	18	10.9	12	9.0	
Had not yet visited	27	9.1	14	8.5	13	9.8	
**Retention in HIV care**							
Adequate	198	66.4	120	72.7	78	58.7	0.010
Inadequate	100	33.6	45	27.3	55	41.3	
***Predisposing factors***							
**Time of diagnosis**							
≤ 12 months prior	96	32.2	63	38.2	33	24.8	0.014
> 12 months prior	202	67.8	102	61.8	100	75.2	
**Had any health problem**^b^	19	6.4	11	6.7	8	6.0	0.819
**Ever diagnosed with TB**	97	32.6	31	18.8	66	49.6	<0.001
**Alcohol drinking**^c^	70	23.5	15	9.1	78	58.7	<0.001
**Illicit drug use**^d^	54	18.1	20	12.1	34	25.6	0.003
**Smoking**^c^	147	49.3	55	33.3	92	69.2	<0.001
**Had unprotected sex**^b^	69	23.2	34	20.6	35	26.3	0.245
**HIV-treatment literacy, mean (SD)**	18.1	(5.29)	19.25	(4.45)	16.74	(5.90)	<0.001
***Enabling factors***							
**Having medical insurance**	74	24.8	31	18.8	43	32.3	0.007
**Connected with HIV-related organization**^e^	120	40.3	56	33.9	64	48.1	0.013
**Mobile-phone use**	286	96	164	99.4	122	91.7	0.001
**SMS use**	283	99	162	98.8	121	99.2	0.743
**Searched HIV information on internet**	168	56.4	134	81.2	34	25.6	0.001
**Internet use (hours)**^f^**, median**	4	-	11	-	0	-	<0.001
***Reinforcing factors***							
**Has been on ART**	168	56.4	84	50.9	84	63.2	0.034
**Having a partner who knows their HIV status**	29	9.7	19	11.5	10	7.5	0.247
**Disclosed HIV status externally**	137	46	65	39.4	72	54.1	0.011
**Experienced stigma & discrimination**^g^	60	20.1	16	9.7	44	33.1	0.001
**Social support, mean (SD)**	3.41	(0.67)	3.22	(0.77)	3.64	(0.42)	<0.001

*Note*: P-values were derived from Chi-Square test for categorical variables, and independent t-tests or Mann-Whitney U test for continuous variables; SD = standard deviation; TB: tuberculosis; ART: antiretroviral therapy;

^a^Waria = Indonesian term for transwomen;

^b^In the past six months^;^

^c^At present;

^d^In lifetime;

^e^Member of/affiliated with;

^f^In the past week;

^g^In the past year.

### Predisposing, enabling, and reinforcing factors

Table 2 also shows the prevalence of predisposing, enabling, and reinforcing factors, as well as differences in these factors between MSM and waria. Regarding predisposing factors, 67.8% of participants were diagnosed with HIV more than 12 months prior to the survey, 6.4% reported having health problems in the past six months, 23.2% had unprotected sex in the past six months. Compared to waria, MSM were less likely to use alcohol, use illicit drugs, smoke, and ever have TB. MSM had a higher HIV-treatment literacy than waria.

Regarding enabling factors, only 24.8% had medical insurance. Compared to waria, MSM were less likely to have medical insurance, to be a member of or affiliated with an HIV-related organization, more likely to use mobile phones, more likely to search for HIV information on the internet, and had higher internet usage.

Regarding reinforcing factors, more than half of all respondents were already on ART (56.4%), with MSM less likely than waria to have been on ART. Compared to waria, MSM were less likely to disclose their HIV status beyond family and partners, were less likely to experience stigma and discrimination, and had lower social support.

### Univariate differences for retention in care

Retention in care was associated with several of the sociodemographic, predisposing, enabling, and reinforcing factors that were considered (Table 3). As noted in Table 2, MSM were more likely to have adequate retention (73%) than waria (59%). Compared to those who had inadequate retention in care, participants with adequate retention had a significantly higher monthly income, and were less likely to have engaged in sex work throughout their lifetime.

**Table 3:** Sociodemographic, predisposing, enabling, and reinforcing factors by retention in care.

			Retention in care				
	Total		Adequate		Inadequate		
Variable	*N*	*%*	*n*	*%*	*n*	*%*	*p-value*
**Total**	298	100	198	66.4	100	33.6	
**Age, mean (SD)**	32.1	(0.43)	32.4	(0.52)	31.7	(0.74)	0.411
**Highest education level**							
Primary school or lower	14	4.7	10	5.1	4	4.0	0.305
Secondary school	63	21.1	36	18.2	27	27.0	
Higher secondary school	173	58.1	121	61.1	52	52.0	
College degree or above	48	16.1	31	15.7	17	17.0	
**Monthly income (in US dollars), median**	109.1	-	136.4	-	90.9	-	0.003
**Relationship status**							
Single	231	77.5	156	78.8	75	75.0	0.449
Has an HIV-negative partner	28	9.4	20	10.1	8	8.0	
Has an HIV-positive partner	16	5.4	10	5.1	6	6.0	
Has a partner with unknown HIV status	23	7.7	12	6.1	11	11.0	
**Area of residence**							
Rural area/small town	61	20.5	38	19.2	23	23.0	0.442
Large town/city	237	79.5	160	80.8	77	77.0	
**Recruitment site**							
Jakarta and surroundings	76	25.5	54	27.3	22	22.0	0.651
Bandung and surroundings	148	49.7	94	47.5	54	54.0	
Yogyakarta & Central Java	46	15.4	30	15.2	16	16.0	
City outside of Java Island	28	9.4	20	10.1	8	8.0	
**Sex work in lifetime**	94	31.5	50	25.3	44	44.0	0.001
**Injection drug use in lifetime**	4	1.3	2	1.0	2	2.0	0.483
***Predisposing factors***							
**Time of diagnosis**							
≤ 12 months prior	96	32.2	63	31.8	33	33.0	0.837
> 12 months prior	202	67.8	135	68.2	67	67.0	
**Had any health problem**^a^	19	6.4	16	8.1	3	3.0	0.090
**Ever diagnosed with TB**	97	32.6	65	32.8	32	32.0	0.885
**Alcohol drinking**^b^	70	23.5	37	18.7	33	33.0	0.006
**Illicit drugs use**^c^	54	18.1	35	17.7	19	19.0	0.779
**Smoking**^b^	147	49.3	83	41.9	64	64.0	<0.001
**Had unprotected sex**^a^	69	23.2	35	17.7	34	34.0	0.002
**HIV-treatment literacy, mean (SD)**	18.1	(0.31)	19.3	(0.32)	15.7	(0.59)	<0.001
***Enabling factors***							
**Having medical insurance**	74	24.8	57	28.8	17	17.0	0.026
**Connected with HIV-related organization**^d^	120	40.3	74	37.4	46	46.0	0.152
**Searched HIV information on internet**	168	56.4	120	60.6	48	48.0	0.038
**Internet use (hours)**^e^**, median**	4	-	5	-	3	-	0.532
***Reinforcing factors***							
**Has been on ART**	168	56.4	135	68.2	33	33.0	<0.001
**Having partner who knows their HIV status**	29	9.7	16	8.1	13	13.0	0.176
**Disclosed HIV status externally**	137	46.0	88	44.4	49	49.0	0.456
**Experienced stigma & discrimination**^f^	60	20.1	27	13.6	33	33.0	<0.001
**Social support, mean (SD)**	3.41	(0.04)	3.35	(0.05)	3.54	(0.06)	0.018

*Note*: P-values were derived from Chi-Square test for categorical variables, and independent t-tests or Mann-Whitney U test for continuous variables; SD = standard deviation; TB: tuberculosis; ART: antiretroviral therapy;

^a^In the past six months^;^

^b^At present;

^c^In lifetime;

^d^Member of/affiliated with;

^e^In the past week;

^f^In the past year.

Compared to those who had inadequate retention in care, participants with adequate retention were less likely to drink alcohol, smoke, and have unprotected sex. They also had higher HIV-treatment literacy. Such participants were also more likely to have medical insurance, more likely to search for HIV information on the internet and more likely to have been on ART. Further they were less likely to experience stigma and discrimination but they also reported having less social support.

### Multivariable associations of retention in care

Model 1 (Table 4) indicates that none of the sociodemographic variables were significantly associated with retention in care. Model 2 shows that after adjusting for sociodemographics, having any health problems (adjusted odds ratio [AOR] = 4.45; 95% confidence interval [CI]: 1.05–18.5) and higher HIV-treatment literacy (AOR = 1.12; 95% CI: 1.10–17.9) were significantly associated with higher odds of adequate retention in care. These two factors remained statistically significant when enabling factors were included in Model 3. Controlling for sociodemographic, predisposing, and enabling factors, being a member of or affiliated with an HIV-related organization was associated with lower odds of retention in care (AOR = 0.50; 95% CI: 0.27–0.91).

**Table 4:** Multivariable associations of sociodemographic characteristics, and predisposing, enabling, and reinforcing factors on adequate retention in care.

Covariate	Model 1	Model 2	Model 3	Model 4
	AOR (95% CI)	AOR (95% CI)	AOR (95% CI)	AOR (95% CI)
***Sociodemographic***				
**Age**	1.03 (0.99–1.07)	1.01 (0.96–1.05)	1.02 (0.97–1.06)	0.99 (0.95–1.05)
**Risk group**				
Waria^a^ (ref)	1.00	1.00	1.00	1.00
MSM	1.68 (0.16–3.70)	1.37 (0.58–3.22)	1.46 (0.59–3.60)	1.26 (0.47–3.40)
**Highest education level**				
Primary school or lower (ref)	1.00	1.00	1.00	1.00
Secondary school	0.75 (0.20–2.76)	1.31 (0.31–5.52)	1.41 (0.33–6.08)	1.85 (0.38–8.96)
Higher secondary school	0.80 (0.22–2.93)	0.96 (0.24–5.52)	0.92 (0.22–3.79)	1.32 (0.28–6.16)
College degree or above	0.38 (0.09–1.63)	0.43 (0.09–2.03)	0.46 (0.09–2.26)	0.84 (0.15–4.75)
**Monthly income (in US dollars)**^b^	1.00 (0.99–1.01)	1.00 (0.09–2.03)	1.00 (0.99–1.00)	1.00 (0.99–1.00)
**Sex worker in lifetime**	0.55 (0.28–1.09)	0.72 (0.34–1.54)	0.81 (0.37–1.77)	0.97 (0.38–2.11)
***Predisposing factors***				
**Had any health problem**^c^	-	4.45 (1.10–17.9)*	4.41 (1.05–18.5)*	2.93 (0.64–13.4)
**Had unsafe sex**^c^	-	0.53 (0.28–1.00)	0.57 (0.30–1.08)	0.72 (0.35–1.49)
**HIV treatment literacy**	-	1.12 (1.06–1.18)***	1.12 (1.06–1.19)***	1.05 (0.99–1.13)
**Alcohol drinking**^d^	-	1.00 (0.47–2.14)	1.04 (0.48–2.24)	1.01 (0.44–2.34)
**Smoking**^d^	-	0.63 (0.34–1.17)	0.66 (0.35–1.23)	0.78 (0.40–1.55)
***Enabling factors***				
**Connected with HIV-related organization**^e^	-	-	0.50 (0.27–0.91)*	0.47 (0.24–0.95)*
**Internet use for HIV information**	-	-	1.26 (0.64–2.48)	2.15 (1.00–4.59)*
**Having medical insurance**	-	-	1.93 (0.94–3.96)	2.84 (1.27–6.34)*
***Reinforcing factors***				
**Has been on ART**	-	-	-	6.00 (2.93–12.3)***
**Having a partner who knows their status**	-	-	-	0.44 (0.16–1.21)
**Ever experienced stigma & discrimination**^f^	-	-	-	0.54 (0.25–1.16)
**Social support**	-	-	-	0.60 (0.34–1.06)

*Note*: OR = Odds Ratio; CI = confidence interval; AOR = Adjusted Odds Ratio;

* p < 0.05;

*** p <0.001;

dashes (-) indicate that the variable was not included in the model;

ref = reference group; ART: antiretroviral therapy;

^a^Waria = Indonesia term for transwomen;

^b^Per $100;

^c^In the past six months;

^d^At present;

^e^Member of/affiliated with;

^f^In the past year.

In Model 4, which added reinforcing factors, having been on ART (AOR = 6.00; 95% CI: 2.93–12.3) was associated with higher odds of adequate retention in care. Respondents who searched for HIV-information on the Internet (AOR = 2.15; 95% CI: 1.00–4.59) and had medical insurance (AOR = 2.84; 95% CI: 1.27–6.34) were also associated with higher odds of retention in care. Being a member of or affiliated with an HIV-related organization remained associated with lower odds of adequate retention in care (AOR = 0.47; 95% CI: 0.24–0.95), whereas having any health problems or higher HIV treatment literacy were no longer associated with retention in care in Model 4.

## Discussion

This study demonstrated high rates of early linkage to care and a moderate rate of adequate retention in HIV care among HIV-positive MSM and waria in Indonesia. Our analysis showed that two predisposing factors, i.e. having other health problems and higher literacy of HIV treatment seem to facilitate to the higher odds of adequate retention in care among our study participants when accounting for sociodemographic characteristics and enabling factors. For the enabling factors, using the internet for HIV information and having medical insurance facilitated adequate retention in care once we controlled for reinforcing factors. Being on ART was the reinforcing factor that could facilitate adequate retention in care. Whereas involvement with HIV-related organizations seemed to inhibit retention in care among our study participants.

The relatively high rates of linkage to care among our study participants is comparable to that among general HIV-positive patients in Indonesia [33], suggesting that there might not be disparities in linkage to care between MSM and waria populations compared to other groups in Indonesia. Also, the prevalence of MSM and waria in our study that have adequate retention in HIV care is similar to the prevalence of retention in care of all HIV-positive individuals in Indonesia taken together [17]. Our results show a higher proportion of adequate retention in care in our sample than HIV-positive MSM and transgender women in other global locations [5, 31, 34, 35].

This is to the best of our knowledge the first study in Indonesia documenting retention in care of HIV-positive MSM and waria and its social aspects. A sizeable number of people from these difficult to reach groups were interviewed, allowing to capture an overall view of retention in care among these groups. Nevertheless, this study also has several limitations. Firstly, the survey used a non-random sampling approach in urban settings, so findings may not be representative of the entire population of MSM and waria groups in Indonesia. The use of targeted sampling which is similar to snowball sampling may also have limited our study participants to certain social network. Secondly, MSM and waria were pooled in the analysis, while descriptive analysis showed that these groups differed on several aspects. The sample sizes, however, were too small to perform subgroup analysis with adequate power. For the same reason we could not stratify participants by time of diagnosis, which might be important because standards of clinical care for people newly diagnosed with HIV may be different from those given to people who have been living with HIV longer [5]. Lastly, this study is subject to social desirability due the nature of self-reported data collection, although all precautions were taken during data collected to reduce this bias to a minimum.

Participants who had higher HIV-treatment literacy and who had other health problems were more likely to be retained in care, but these associations lost significance when taking ART status into account. It is common that HIV patients seek care only when they experience symptoms of disease [36], and our results appear to confirm this tendency that participants who are in good health do not seek health care regularly. Our finding also supports the notion that sufficient literacy or knowledge about the benefits of HIV treatment could increase motivation to seek and remain in treatment. To our knowledge, prior studies have not examined the association between knowledge and retention in care, although some studies have shown that a lack of knowledge about HIV treatment is a barrier to HIV testing [37] and ART use [38].

Knowledge about HIV care and treatment may be obtained by searching HIV-related materials through any source of information, including the internet. Our results show a positive association between using the internet to look for HIV information and adequate retention in care. This finding supports prior studies that identified the strong potential of internet use for HIV-positive individuals in the HIV care continuum [39–42]. Our study further revealed that medical insurance predicted greater odds of retention in HIV care. This implies that financial reasons may prevent people with HIV in Indonesia from routinely accessing HIV care. In lower-middle income countries, a fee-for-service program was associated with a lower probability that people with HIV will continue treatment after ART initiation [43]. Likewise, a systematic review showed that having private insurance was associated with higher utilization rates of health services among people with HIV, even in high income countries [44].

A surprising finding in our study is that participants who are attached to an HIV-related organization are less likely to be retained in care. We assumed such attachment could bring support for people with HIV, in turn enabling them to be retained in care. HIV-related organizations usually have solid networking with local HIV care and treatment services [45, 46], and thus would encourage people with HIV to be adequately retained in care [5]. Nevertheless, our findings indicate that having such support may possibly keep them away from health care. Maintaining a healthy status to keep the doctor away is a basic health concept among Indonesian people for financial reasons. It is possible that participants with support from HIV-related organizations perceive themselves as being in good health and find it unnecessary to visit HIV care too often [47]. More insight into this phenomenon is needed in order to improve retention to care among these individuals.

As expected, we found that ART status had a positive association with retention in care. This aligns with other studies indicating a positive association between ART initiation with retention in care [48], and with a higher utilization of health services [44, 49]. Our analysis further showed that once HIV-positive MSM and waria are on ART, predisposing factors are no longer influential towards retention in care. In Indonesia, people on ART are required to visit an HIV care facility every month for a one-month supply of ART pills. A buffer stock will only be given if they have an acceptable reason e.g., going out of town. This strategy may encourage people with HIV to visit HIV care regularly.

Our findings suggest that it is important to ensure that adequate information on HIV care is available online [39, 42]. We identified a large proportion of internet users among our participants, particularly among MSM. Internet should thus be considered as one alternative medium to improve adherence. In view of the high rate of mobile phone use, future research on the efficacy of mobile phone based interventions [50], either through text messaging [51–54] or smart-phone applications [55] is worth considering. It is also important to guarantee that HIV care is affordable for people with HIV. It is crucial to lower the cost of HIV care or to give people with HIV access to a health insurance program. Being on ART gives a greater likelihood that people with HIV remain in HIV care, therefore a strategy seems appropriate that puts newly diagnosed MSM and waria on ART immediately [56, 57]. Lastly, more knowledge is needed to comprehend how organizational support influences retention in care of people with HIV in Indonesia.

## Conclusions

Our results fill gaps on data about two crucial stages in the HIV treatment cascade, i.e., linkage to and retention in care, specifically for HIV-positive MSM and transgender women in Indonesia. This study describes how sociodemographic characteristics and social determinants influence retention in care. As highlighted in our study, future interventions should carefully consider socioeconomic and cultural barriers and use internet-based technology to improve retention in care, and ultimately viral load suppression in these two vulnerable populations.
